# Computed tomography findings in a cohort of 169 dogs with elbow dysplasia - a retrospective study

**DOI:** 10.1186/s12917-021-02997-5

**Published:** 2021-09-06

**Authors:** Mateusz Hebel, Wojciech K. Panek, Jakub J. Ruszkowski, Maria Nabzdyk, Dariusz Niedzielski, Katarzyna C. Pituch, Aaron M. Jackson, Maciej Kiełbowicz, Małgorzata Pomorska-Mól

**Affiliations:** 1 Department of Internal Diseases and Diagnostics, Poznan University of Life Sciences, ul. Wołyńska 35, 60-637 Poznań, Poland; 2 Department of Clinical Sciences, College of Veterinary Medicine, North Carolina State University, Raleigh, North Carolina 27607 USA; 3 Department of Animal Anatomy, Poznan University of Life Sciences, ul. Wojska Polskiego 71C, 60-625 Poznań, Poland; 4 University Centre for Veterinary Medicine, Szydłowska 43, 60-656 Poznan, Poland; 5 Small Animal Veterinary Clinic, Klinika Psa i Kota, ul. Bolesława Krzywoustego 105/21, 51-166 Wrocław, Poland; 6 Department of Neurological Surgery, Feinberg School of Med, 676 N St. Clair, Suite 2210, Chicago, IL 60611 USA; 7 Department of Small Animal Surgery, Medvet Chicago, 3123 N. Clybourn Ave, Chicago, IL 60618 USA; 8 Department of Preclinical Sciences and Infectious Diseases, Poznan University of Life Sciences, ul. Wołyńska 35, 60-637 Poznań, Poland

**Keywords:** Canine elbow dysplasia, CED, CT, Imaging, Dogs

## Abstract

**Background:**

Canine elbow dysplasia (CED) is a complex developmental skeletal disorder associated with a number of pathological conditions within the cubital joint. Because CED is a heritable disease, it is important to identify and remove the affected animals from breeding. The first objective of this study was to describe the prevalence of medial coronoid process disease (MCPD) without (MCD) or with (FMCP) fragmented medial coronoid process, osteochondrosis (OC) and/or osteochondritis dissecans (OCD), ununited anconeal process (UAP), radio-ulnar incongruence (INC R-U) and humero-ulnar incongruence (INC H-U) in dogs with the use of CT imaging. The second aim was to determine the influence of demographics on the prevalence of investigated pathologies in dogs with clinical evidence of elbow dysplasia.

**Results:**

In this retrospective study, CT data records of 169 dogs of different breeds presented to the small animal veterinary clinic from 2012 to 2018 were included. 69.23% of dogs diagnosed with CED were young (≤ 2 years old). The mean age of dogs presented with INC R-U was 1.68 ± 1.82 years, while in dogs without INC R-U the mean age was 2.64 ± 2.59 years. The mean age of dogs with INC H-U was 1.94 ± 2.06 years, while without INC H-U 3.29 ± 2.09 years. Labrador Retrievers, German Shepherd and Bernese Mountain dogs were most frequently presented with CED-associated lameness. In 122 dogs OA of varying severity was found.

**Conclusion:**

INC H-U, FMCP and MCD were among the most frequently found components of CED found in the present study. OCD and UAP were the least frequently diagnosed. Dogs presented with INC R-U and INC H-U were significantly younger than dogs without these CED components. Boxers, Dog de Bordeaux, American Staffordshire terriers and mixed-breed dogs were diagnosed later in life than the other breeds. OA of varying severity was found in 72.18% of dogs. Males accounted for more than 75% of the study population.

## Background

Canine elbow dysplasia (CED) is a prevalent health issue that affects many breeds, particularly medium to large sized dogs, however it is also reported in smaller chondrodystrophic breeds like Dachshund and French bulldog [1–3]. Elbow dysplasia is relatively common in dogs with the reported prevalence in animals presented for breed screening up to 70% in Bernese mountain dogs [4], 17.0–29.6% in Labradors [5–7], 26% in Newfoundlands [8]. Several research reports confirmed that certain breeds tend to be affected by a particular entity more frequently than others [2, 9].

CED is a complex developmental skeletal disorder associated with a number of pathological conditions within the cubital joint [1, 10, 11]. Complex primary conditions associated with elbow dysplasia included medial coronoid process disease (MCPD), osteochondrosis (OC) and/or osteochondritis dissecans (OCD), ununited anconeal process (UAP), and joint incongruency (INC) and may be identified separately as a singular cause of elbow dysplasia or occur as a combination of lesions presented simultaneously [1, 12]. Due to the changes observed in the CT image, the MCPD was divided into two groups: without fragmented medial coronoid process (MCD) and with fragmented medial coronoid process (FMCP).

Elbow dysplasia can cause lameness, and arthroscopic treatments do not palliate pain in all affected dogs [13, 14]. Because CED is a heritable disease, it is very important to identify and remove the affected animals from breeding to decrease the incidence of CED in dogs. Although some lesions, including UAP and OCD of the humerus, are quite often successfully identified on plain radiographs, appropriate diagnosis of the MCD or FMCP may be less straight-forward due to superimposition of medial epicondyle and muscle tissue [15]. Computed tomographic imaging is recognized as a high-sensitivity tool that allows for a detailed visualization of the skeletal components of the joints [12, 16–18]. Moreover, computer tomography (CT) enables not only detection but also monitoring of progression in elbow dysplasia [17]. Recently, the use of CT has become increasingly widespread in veterinary medicine. Current data indicate that some components of CED (i.e. MCPD) might be detected as early as 14 weeks of age [19, 20]. Data from previous report indicated that CT should be used when investigating elbow joint diseases in young dogs [19]. However, the number of CT - based studies assessing prevalence of different CED lesions in dogs, including dogs of different breeds is limited [21, 22]. Evaluation of correlations between certain components of CED and specific demographic and/or phenotypic features may allow for more appropriate research in the future and ultimately lead to the identification of new risk factors [23].

The first objective of this study was to evaluate the prevalence of MCPD (MCD and FMCP), OC and/or OCD, UAP, radio-ulnar incongruence (INC R-U) and humero-ulnar incongruence (INC H-U) in dogs with the use of CT imaging. The second aim was to determine the influence of demographics (breed, age, sex) on the prevalence of investigated pathologies in dogs with clinical evidence of elbow dysplasia.

## Methods

### Animals

The study was conducted on CT data records of 169 dogs of different breeds admitted to the small animal veterinary clinic (Klinika Psa i Kota, Wrocław, Poland) from February 2012 to November 2018. Dogs were referred for diagnostic CT imaging due to bilateral or unilateral elbow lameness detected on the basis of a clinical examination conducted by an orthopedic specialist. Only dogs diagnosed with CED were included in the study. Inclusion criteria for study population were: 1) presenting complaint of elbow lameness and CED components in CT image 2) complete information on demographic feature (breed, age, body weight) 3) clinical and orthopedic examination; 4) diagnostic quality CT scans. The exclusion criteria were non-CED associated elbow lameness and incomplete medical records. Data collected from the medical records included: clinical signs (bilateral or unilateral elbow lameness), age and body weight at admission, diagnostic procedures performed.

### Study design

For each dog the following data were retrospectively gathered: breed, age (at the time of first diagnosis), sex and weight. 169 dogs with CED were divided into three different weight categories: medium (15–25 kg, *n* = 16), large (26–44 kg, *n* = 122) and extra-large (≥45 kg, *n* = 31)*.*

All dogs underwent physical and orthopedic examinations followed by a complete blood count and serum biochemistry tests. All study participants were able to walk without assistance. The assessment of mobility function and presence of lameness was performed by one orthopedic specialist veterinary surgeon. CT scans were assessed by two veterinary radiology specialists.

Owners obtained a written description of the study and they provided written informed consent for the inclusion of their dogs in the study.

### CT imaging and analysis

The prevalence of UAP, OCD, MCD, FMCP, INC R-U and INC H-U in dogs with CED was estimated on the basis of the results of CT imaging**.** MCD and FMCP lesions were analyzed separately. The degree of OA was assessed based on International Elbow Work Group (IEWG) guidelines [24]. A total of 338 elbow joints were screened with CT-imaging.

CT images were obtained with a 2-slice helical scanner (Twin, Elscint, Israel) or 16-slice helical scanner (Somatom, Siemens. Germany) using 120 kVp or 110 kVp, 100 mA, pitch 0.8 and reconstructed slices were 0.6 mm with an overlapping slice index of 0.5 mm. Dogs were sedated with intramuscular injection *(i.m)* of medetomidine in a dose of 5–20 μg (micrograms)/kg and butorphanol (0.1–0.4 mg/kg)*.* Induction was obtained with propofol in a dose of 3.2 mg/kg of body weight administered intravenously followed by endotracheal tube placement. Isoflurane and 100% oxygen were used for anesthesia maintenance. During the CT scan dogs were placed in sternal recumbency with both thoracic limbs extended cranially and head was shifted out of the gantry to avoid potential artefacts. DICOM files of each scan were analyzed by an experienced veterinary radiologist using 3D MPR tool in OsiriX (64-bit software, Pixmeo, Geneva, Switzerland) to evaluate the scans in sagittal and dorsal planes.

The level of OA in each elbow joint was scored according to IEWG [24] and the following scores were assigned: 0 - normal elbow joint (no evidence of incongruity, sclerosis or arthrosis); 1- mild arthrosis or suspect primary lesion (presence of osteophytes < 2 mm, sclerosis of the base of the coronoid processes and subtrochlear ulnar region - trabecular pattern still visible); 2- moderate arthrosis (presence of osteophytes 2–5 mm, obvious sclerosis (no trabecular pattern) of the base of the coronoid processes, step of 3–5 mm between radius and ulna (incongruity), indirect signs for other primary lesion (UAP, FMCP/coronoid disease, OCD); 3- severe arthrosis or evident primary lesion (presence of osteophytes > 5 mm, step of > 5 mm between radius and ulna (obvious incongruity), obvious presence of a primary lesion (UAP*,* FMCP, OCD*)* [8].

**Fig. 1:**
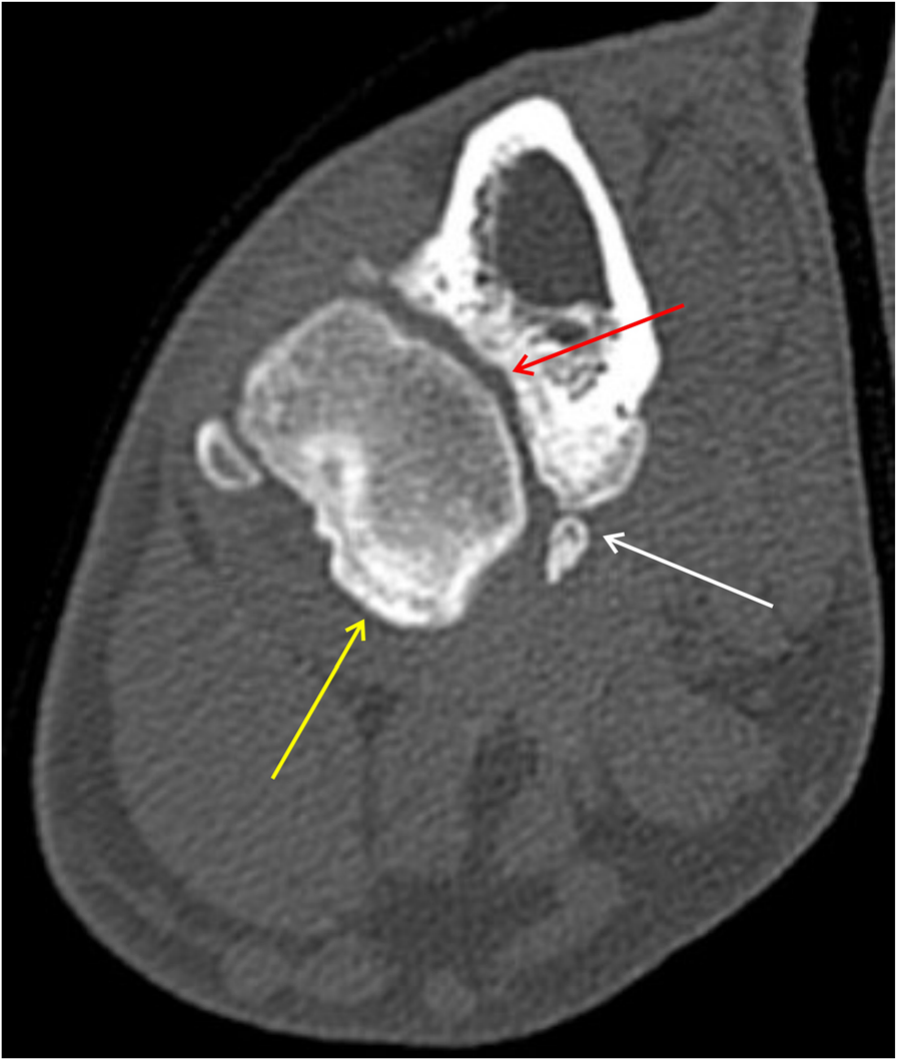
Sagittal MPR image was established via alignment of external borders of humerus and ulna on a dorsal plane (red lines)

**Fig. 2:**
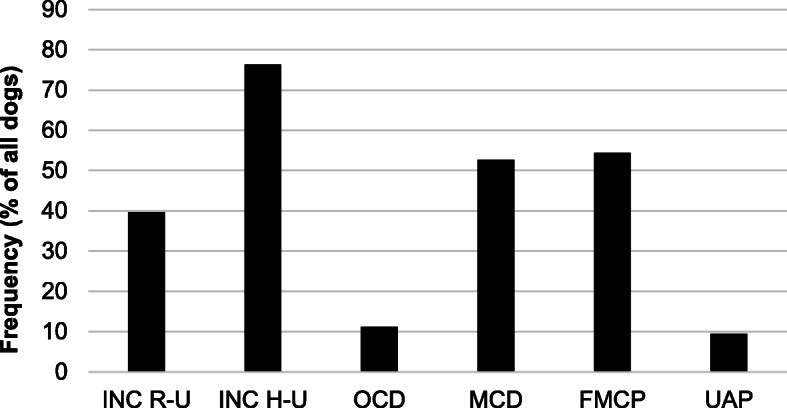
Sagittal and dorsal plane MPR reconstructions (A and B) were used for the evaluation of radio-ulnar incongruence

Assessment of joint incongruency was done using multiplanar reconstructions (MPR) in the mid-coronoid region in an oblique plane, according to scheme presented previously [25, 26]. Sagittal MPR image was established via alignment of external borders of humerus and ulna on a dorsal plane (red lines) (Fig. 1). The reformatted sagittal plane was used to measure incongruence at the base of the coronoid and reformatted dorsal planes were used to assess incongruence at the mid and cranial coronoid regions. Sagittal and dorsal plane MPR reconstructions (A and B) were used for the evaluation of radio-ulnar incongruence (Fig. 2). INC-RU was defined as the distance between the sub-chondral bone surfaces of the radioulnar articulation at the caudal ulnar incisure, mid-body, and cranial apex. Joints with a reduction of the radial articular surface in relation to the ulna by 1.6 mm and more were considered as affected with INC-RU [25, 27].

**Fig. 3:**
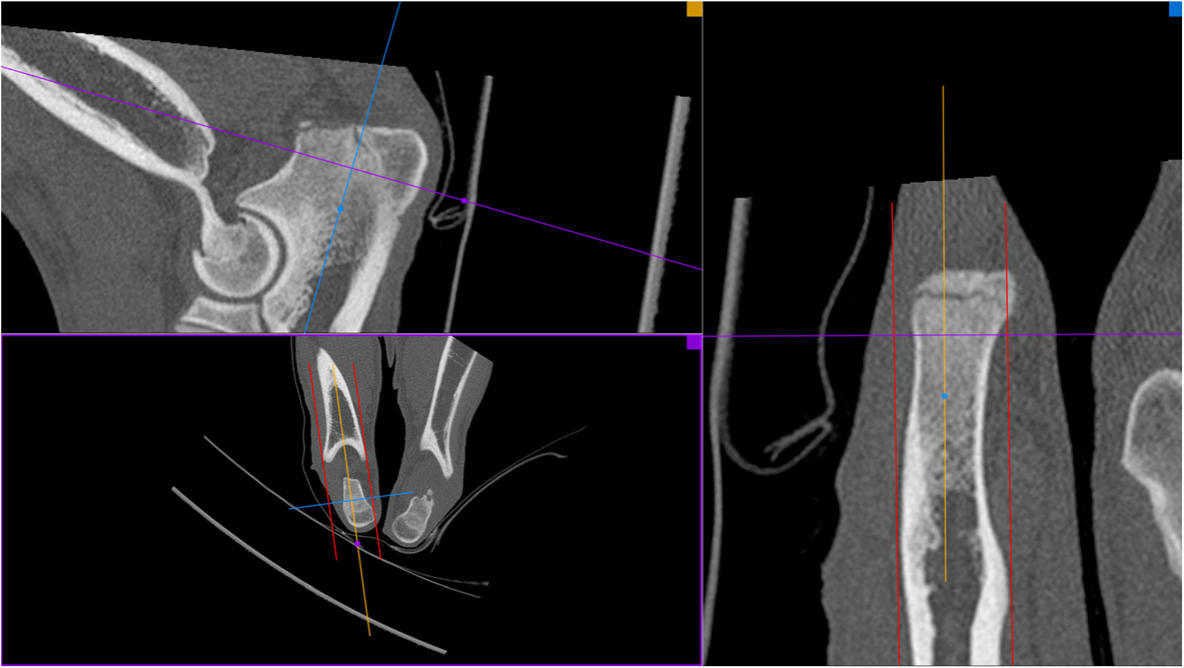
Sagittal CT slice (MPR reconstruction), showing three ulnar measurement loci (distances measured between green lines) for the assessment of humero-ulnar incongruence (INC H-U). Same slice has been used to evaluate ununited anconeal process (UAP), (borders marked with red dotted line) [25, 29]

Humero-ulnar incongruency was subjectively evaluated based on the width of the joint space at the greatest caudal convexity of the trochlear notch of the ulna [28]. The sagittal CT slice (MPR reconstruction), showing three ulnar measurement loci (distances measured between green lines) for the assessment of INC H-U is presented in Fig. 3. Same slice has been used to evaluate UAP, (borders marked with red dotted line).

**Fig. 4:**
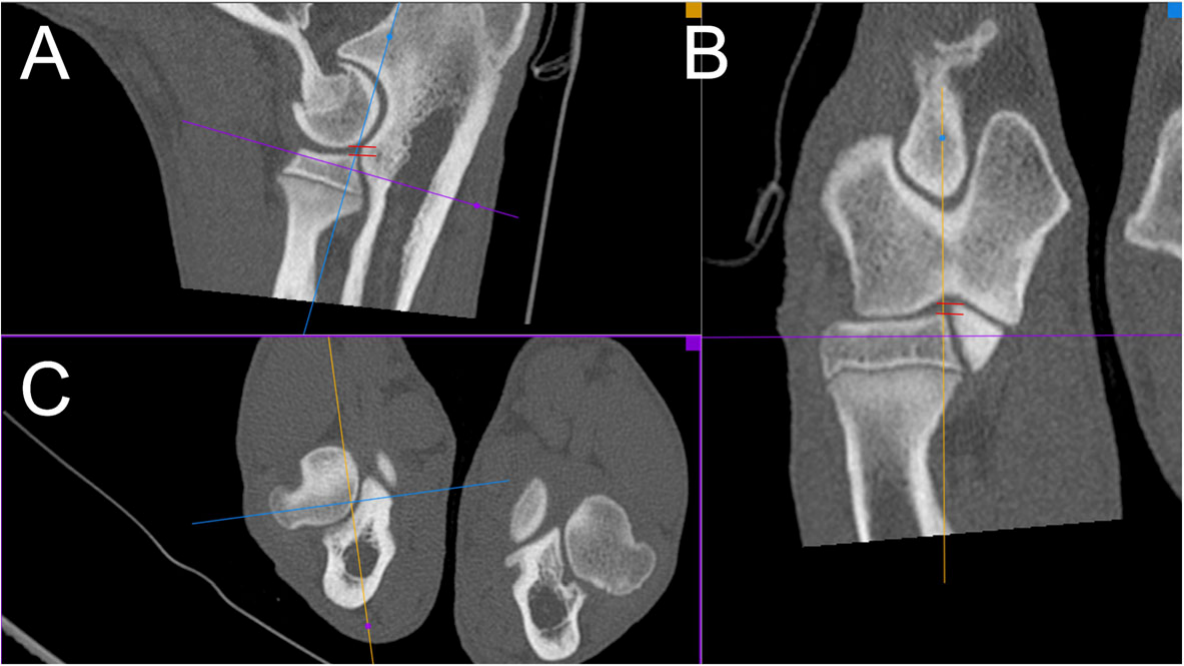
Transverse plane MPR reconstruction (C) at the level of medial coronoid process of the ulna (borders marked with red line) was used for the assessment of MCP pathology [29]

Transverse plane MPR reconstruction (C) at the level of medial coronoid process of the ulna (borders marked with red line) was used for the assessment of MCPD pathology (Fig. 4).

In the present study, focal marked sclerosis causing thickening of the subchondral plate was diagnosed as „kissing lesion”, while a radiolucent subchondral defect on the distal medial trochlea was diagnosed as OCD.

**Fig. 5:**
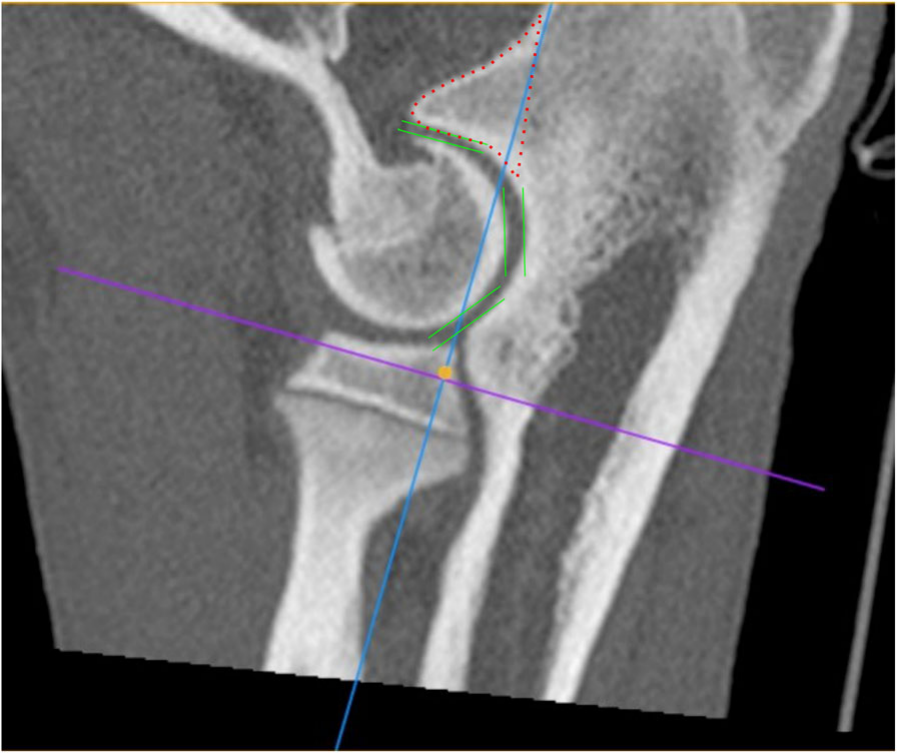
MPR reformatted images in sagittal plane. Yellow arrow shows humero-ulnar incongruity. Green arrow shows subchondral bone sclerosis in trochlear notch of ulnar bone. Red arrow shows osteophytes on the dorsal border of the anconeal process [25, 29]

**Fig. 6:**
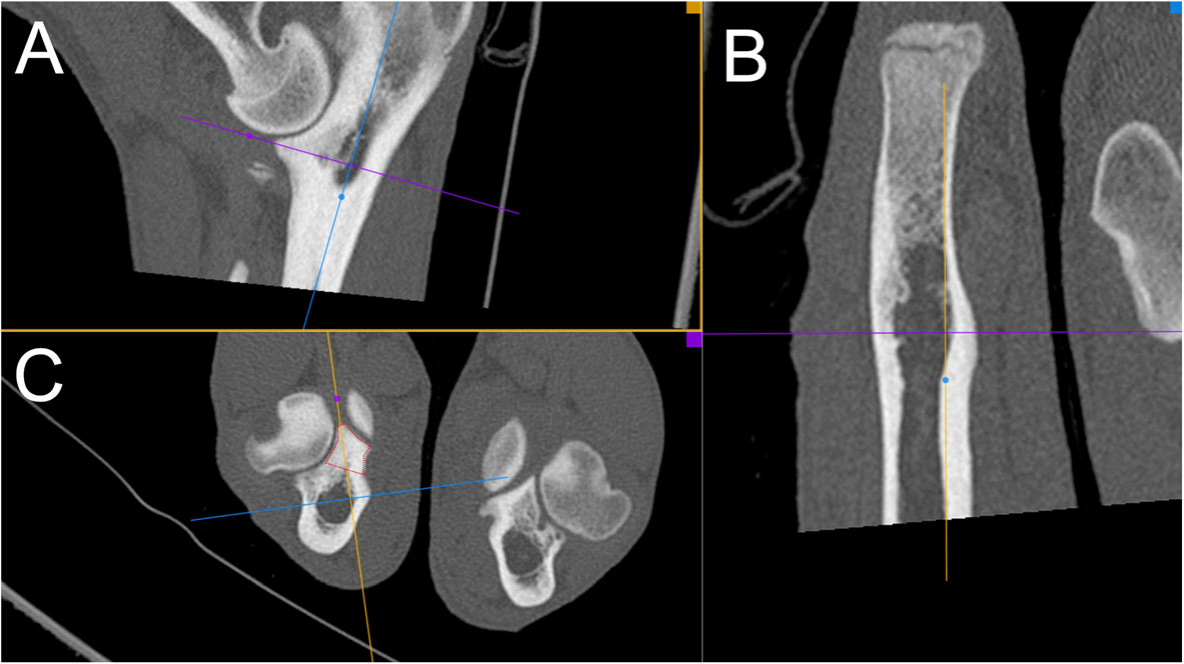
MPR reformatted images in dorsal plane. Space between yellow line shows radioulnar incongruity [25, 29]

**Fig. 7:**
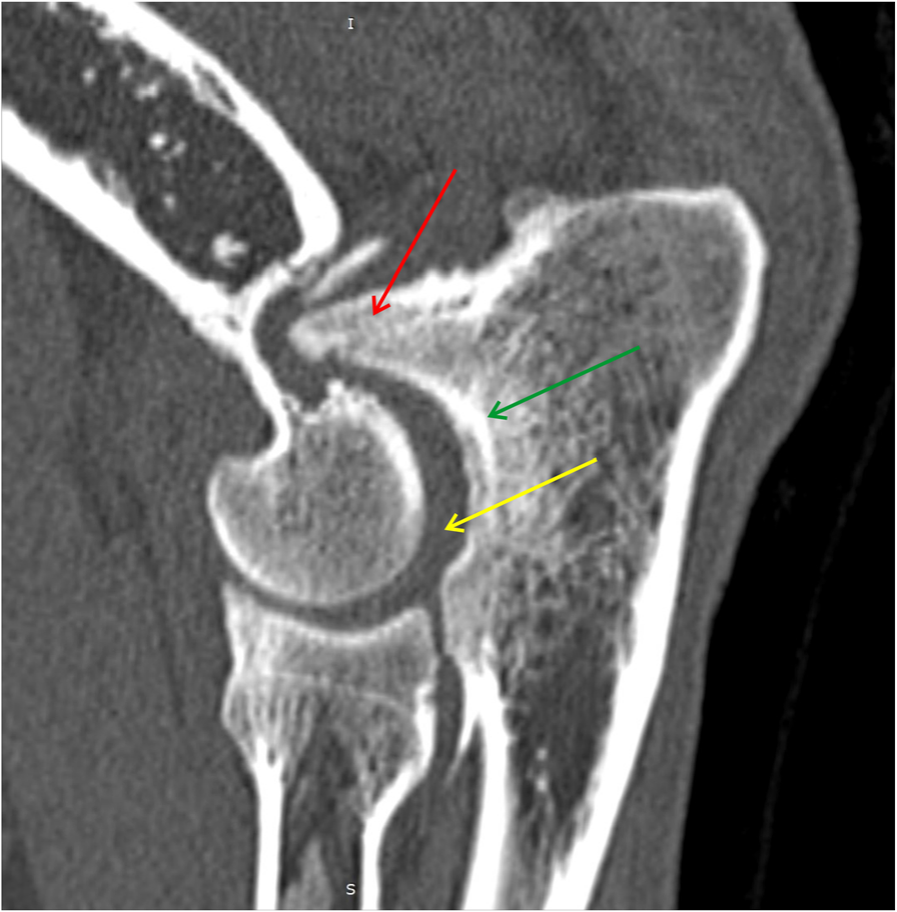
MPR reformatted images in sagittal plane. Space between yellow line shows radioulnar incongruity. Red arrow shows humero-ulnar incongruity. Green arrow shows osteophytes on the dorsal border of the anconeal process [25, 29]

**Fig. 8:**
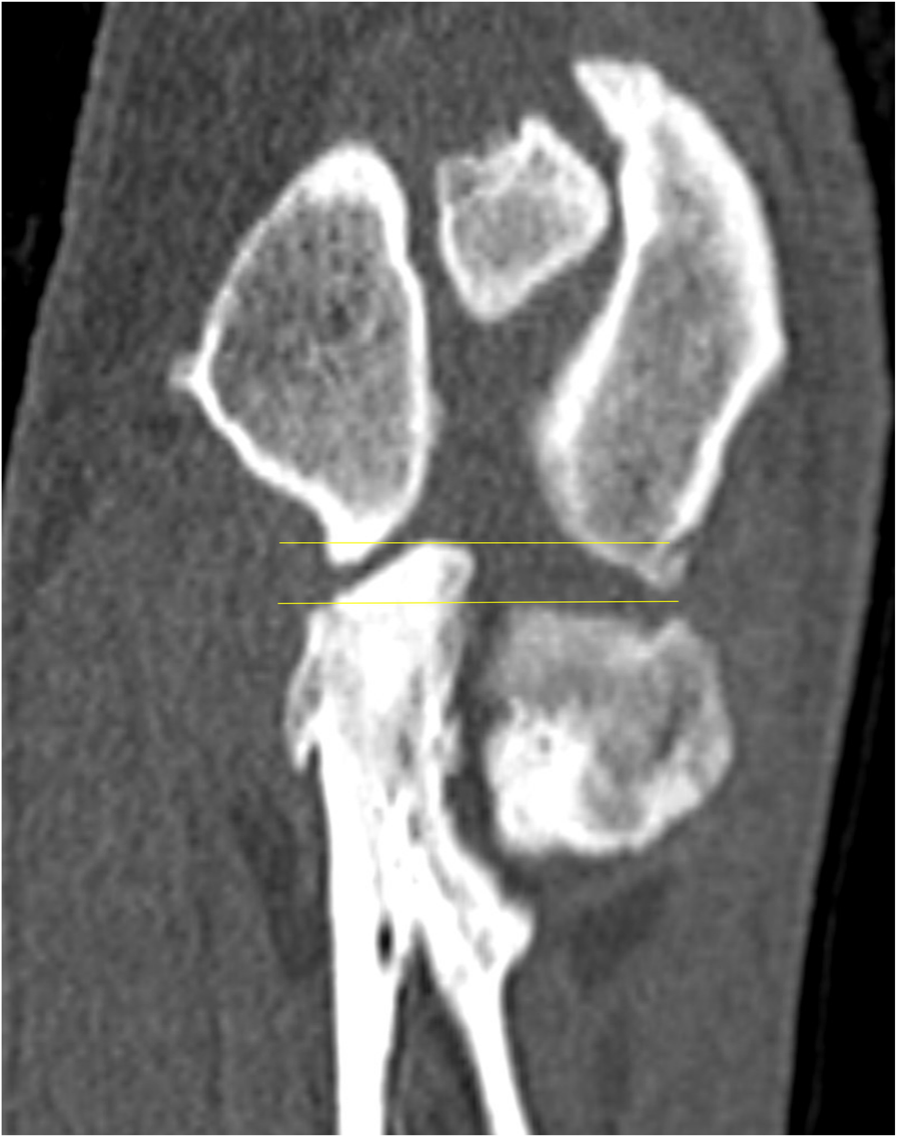
MPR reformatted images in sagittal plane. Red arrow shows humero-ulnar incongruity and subchondral bone sclerosis in trochlear notch of ulnar bone. Green arrow shows ununited anconeal process (UAP) [25, 29]

**Fig. 9:**
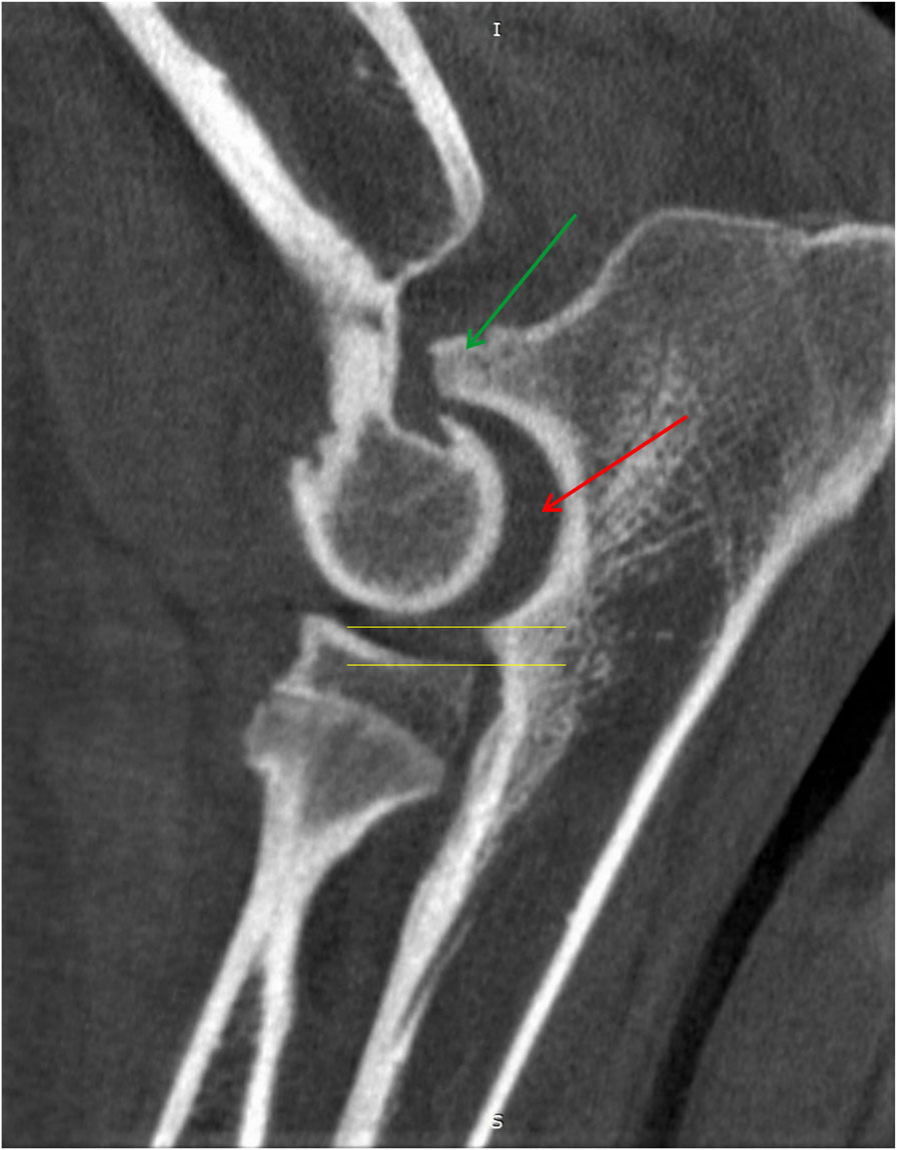
MPR reformatted images in sagittal plane. Red arrow shows defect of the subchondral bone surface of the humeral trochlea, with local subchondral bone sclerosis – OCD. Yellow arrow shows fragmented medial coronoid process (FMCP)

**Fig. 10:**
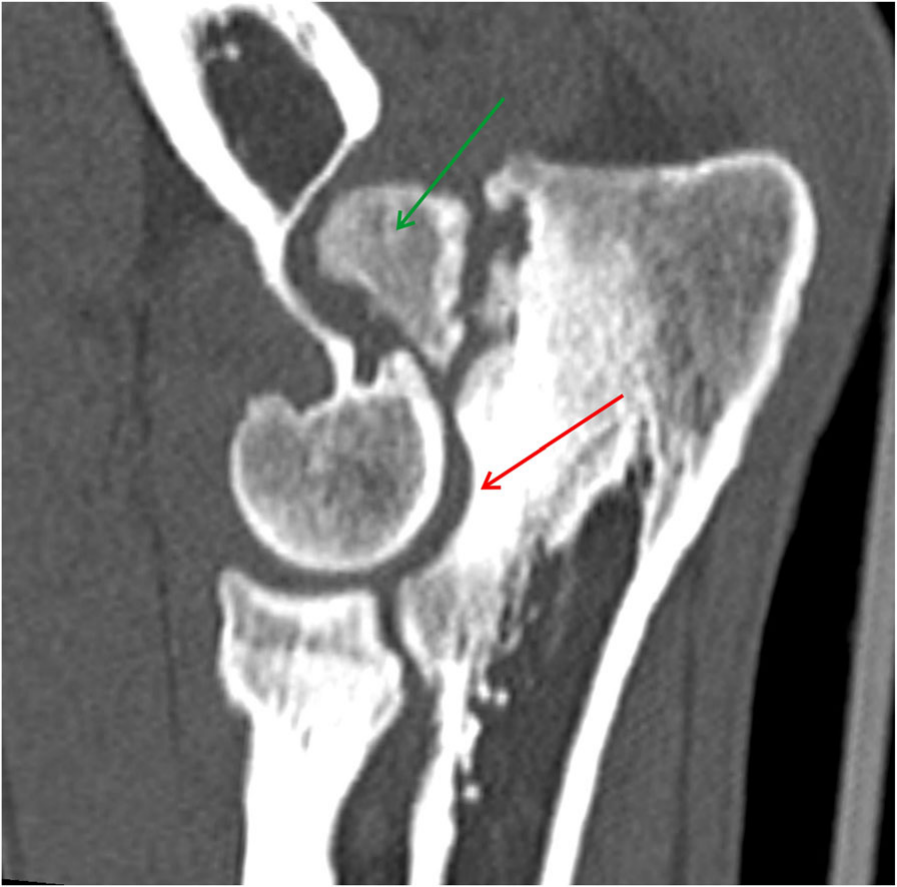
Axial scan on the level of the medial coronoid process of the ulnar bone. White arrow shows inhomogeneous medial coronoid process with small osteophyte on his apex, what suggests medial coronoid process disease

**Fig. 11:**
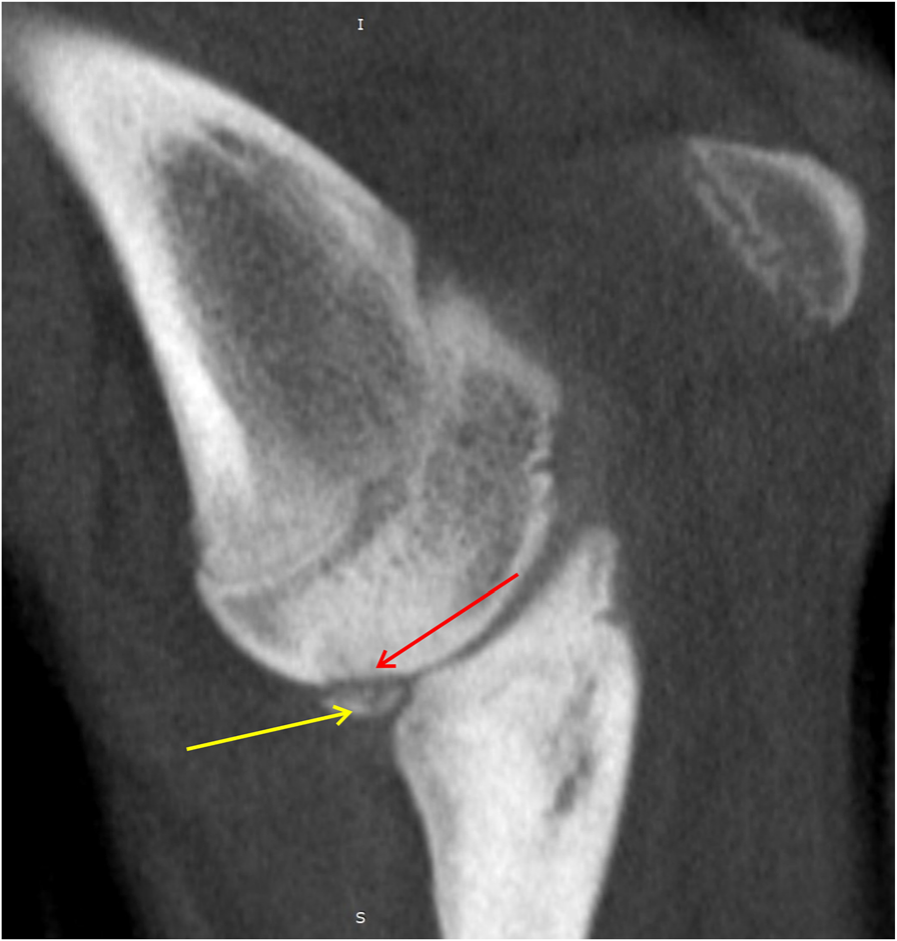
Axial scan on the level of the medial coronoid process of the ulnar bone. White arrow shows fragmented medial coronoid process with small osteophyte on border. Red arrow shows irregular surface of the subchondral bone of the radial notch of ulna. Yellow arrow shows osteophytes on the cranial border of the radial head

The typical changes observed in the present study are presented in Figs. 5, 6, 7, 8, 9, 10, 11.

### Statistical analysis

The obtained data were subjected to the W. Shapiro-Wilk test for normality and the Levene’s test for equality of variances. Differences between means (the mean age of CED affected dogs in particular weight category groups) were tested for statistical significance by a nonparametric Kruskal-Wallis test with post hoc multiple comparisons for comparison of all pairs. For analysis of relationship between the occurrence of various CED component and demographic features (age and body weight) of the tested dogs the U Mann-Whitney test were used. Differences were considered as significant with α < 0.05. All calculations were performed with Statistica 13.3 (Tibco, USA).

## Results

### Dogs characteristics

169 dogs of various breeds met the inclusion criteria (41 females and 128 males). Thirty-one pure breeds were represented. In addition, two mixed-breed dogs were included. The dogs’ ages ranged from 5 months to 10.0 years (median 1 year) and body weights from 15.0 to 70.0 kg (median 36.0 kg). Detailed characteristics and demographic features of each dog are presented in Table 1. Sixteen dogs were classified to medium weight category, 122 to large weight category, and 31 to extra-large weight category. Detailed characteristics of demographic features in three weight categories are presented in Tables 2 and 3.

**Table 1:** Characteristics of dogs included in the study. Data were collected from 169 dogs with canine elbow dysplasia

Breed	Number of included dogs	Median age years (range)	Sex (number) males/females	Median weight kg (range)
Labrador Retriever	43	1.5 (0.4–10)	36/7	35.16 (25–47)
German Shepherd Dog	40	0.91 (0.4–9.0)	29/11	36.74 (29–50)
Bernese Mountain dog	15	1 (0.5–6.5)	12/3	42.4 (33–55)
Chow Chow	6	1.04 (0.4–3.0)	4/2	22.5 (15–28)
Boxer	5	6 (2.0–9.0)	2/3	33 (19–40)
Golden Retriever	5	0.58 (0.4–3.5)	3/2	28.6 (19–36)
Dog de Bordeaux	5	3.5 (0.5–6.0)	3/2	52.4 (27–70)
Cane Corso	5	0.83 (0.4–3.0)	4/1	39.8 (26–50)
White Swiss Shepherd	4	0.7 (0.5–1.0)	4/0	31 (28–33)
Rottweiler	4	1.4 (0.5–6.0)	3/1	44 (39–52)
American Staffordshire Terrier	3	4.75 (1–10)	3/0	30.3 (29–32)
Polish Tatra Sheepdog	3	0.66 (0.58–3.0)	2/1	44.3 (32–61)
Bouvier des Flandres	3	0.83 (0.4–3.0)	2/1	37.3 (35–41)
English Bulldog	3	0.83 (0.8–3.0)	3/0	33.3 (24–44)
Mixed-breed	2	3 & 7.5	2/0	37 & 35
Newfoundland	2	0.66 & 1.0	2/0	61 & 60
Caucasian Shepherd	2	0.4 & 1.5	2/0	20 & 50
Eurasian Shepherd	2	0.5 & 2.0	1/1	47 & 68
Pitbull	2	0.4 & 0.5	1/1	20 & 25
Argentinian Mastiff	2	1 & 3.0	2/0	58 & 59
Border Collie	1	1.5	1/0	23
Bullmastiff	1	0.66	1/0	50
Australian Cattle dog	1	2.0	0/1	23
Beagle	1	5.0	1/0	23
St. Bernard	1	0.5	1/0	40
American Bully	1	0.58	1/0	26
Great Dane	1	1.5	0/1	36
Greater Swiss Mountain	1	1.0	1/0	51
Flat Coated Retriever	1	1.0	1/0	30
Spanish Mastiff	1	0.75	0/1	60
Shar-pei	1	1.0	0/1	25
Giant Schnauzer	1	1.5	1/0	42
Spanish Alano	1	0.5	0/1	52

**Table 2:** Descriptive statistics of study population: body weight (kg)

Weight category	Body weight (kg)					
	n	Mean	Median	Minimum	Maximum	SD
Medium	16	21.87	23.00	15.00	25.00	3.05
Large	122	35.24	35.00	26.00	44.00	4.90
Extra-large	31	52.80	50.00	45.00	70.00	6.74

*n* number of dogs; *SD* standard deviation

**Table 3:** Descriptive statistics of study population: age (years)

Weight category	Age (years)					
	n	Mean	Median	Minimum	Maximum	SD
Medium	16	1.14	0.58	0.41	6.00	1.40
Large	122	2.41	1.00	0.41	10.00	2.56
Extra-large	31	2.27	1.50	0.50	6.50	1.80

*n* number of dogs; *SD* standard deviation

The significant differences have been found between age of dogs affected and not affected with INC R-U and INC H-U (*p* = 0.02 and *p* = 0.01, respectively). The mean age of dogs with INC R-U was 1.68 ± 1.82 years (with a median of 0.83), whereas in dogs without this component of CED the mean age was 2.64 ± 2.59 years (with a median of 1.5). Similar results were obtained with INC H-U. The mean age of dogs with INC H-U was 1.94 ± 2.06 years (with a median age of 1 year), whereas in dogs affected by CED but without INC H-U, the mean age was 3.29 ± 2.09 years (with a median age of 2 years). For the other components of CED (OCD, MCD, FMCP and UAP), no age-related differences were found between dogs with and without the pathology studied (*p* ≥ 0.05).

A more detailed analysis showed that in the youngest dogs (up to 6 months), INC H-U and/or FMCP were the most common pathologies. In dogs aged between 6 months and 2 years, INC H-U and/or FMCP were also most frequently found. In older dogs, aged between 3 and 6 years, the INC H-U, MCD and/or FMPC were the most common. In the oldest dogs, aged over 6 years, INC H-U and MCD were the most often observed. Details of the occurrence of the investigated pathologies in dogs of different ages are shown in Table 4.

**Table 4:**
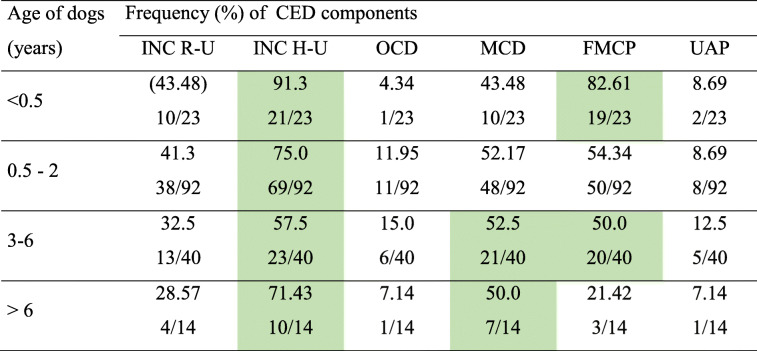
The frequency (%) and absolute number of dogs affected with various components of CED depending on age category

*n* number of dogs; the most common finding in each age category is marked in green. *CED* canine elbow dysplasia; *INC R-U* radio-ulnar incongruence; *INC H-U* humero-ulnar incongruence; *FMCP* medial coronoid disease with fragmented medial coronoid process; *OCD* osteochondrosis dissecans within the medial compartment of humeral condyle coronoid process; *MCD* medial coronoid disease without fragmented medial coronoid process (pathological cartilage and or subchondral bone); *UAP* ununited anconeal process

No significant differences were found for the mean body weight of dogs with or without each investigated pathology (INC R-U, INC H-U, OCD, MCD, FMCP and UAP) (*p* ≥ 0.05).

In dogs from medium weight category the most common pathologies were INC H-U (9/16) and FMCP (8/16), while UAP was observed only in 2 out of 16 dogs. In dogs from large weight category INC H-U was found in 90/122, while FMCP and MCD were found in 66 and 63 dogs out of 122, respectively. The OCD and UAP were found the least frequently (in 11 and 13 dogs, respectively). In the extra-large category the most common finding were INC H-U (26/32) and MCD (19/32), while UAP was found in only 4 out of 32 dogs. The percentage of dogs with various components of CED in particular weight category is presented in Table 5.

**Table 5:** The frequency (%) and absolute number of dogs affected with various components of CED depending on weight category

Weight category	Frequency (%) of CED components					
	INC R-U	INC H-U	OCD	MCD	FMCP	UAP
Medium	(37.50) 6/16	(56.25) 9/16	(18.75) 3/16	(31.25) 5/16	(50.00) 8/16	(12.50) 2/16
Large	(40.98) 50/122	(73.77) 90/122	(9.02) 11/122	(51.64) 63/122	(54.09) 66/122	(10.65) 13/122
Extra-large	(38.71) 12/31	(83.87) 26/31	(19.35) 6/31	(61.29) 19/31	(38.71) 12/31	(12.90) 4/31

*CED* canine elbow dysplasia; *INC R-U* radio-ulnar incongruence; *INC H-U* humero-ulnar incongruence; *FMCP* medial coronoid disease with fragmented medial coronoid process; *OCD* osteochondrosis dissecans within the medial compartment of humeral condyle coronoid process; *MCD* medial coronoid disease without fragmented medial coronoid process (pathological cartilage and or subchondral bone); *UAP* ununited anconeal process

Labrador Retrievers (43%), German Shepherd dogs (GSD) (40%) and Bernese Mountain dogs (15%) were most frequently presented with CED-associated lameness. In addition, patients belonging to these breeds represented the majority of the subjects within the cohort of large-size dogs, with weights ranging between 29.0 and 50.0 kg for GSD, 20.5 and 47.0 kg for Labrador Retrievers, and 33.0 and 50.5 kg for Bernese Mountain dogs.

In 47 dogs no signs of OA were observed on CT, while in the other 122 animals OA of varying severity was found. Within dogs with OA, 57/122 (46.72%) were assigned a grade 1; 27/122 (22.13%) were assigned a grade 2; 38/122 (31.14%) were assigned a grade 3. It is worth to mention that dogs with a different degree of OA in each joint were classified to the group corresponding to the higher degree of OA. 11.6% of dogs were diagnosed with unilateral CED and 88.4% suffered from bilateral CED.

**Fig. 12:**
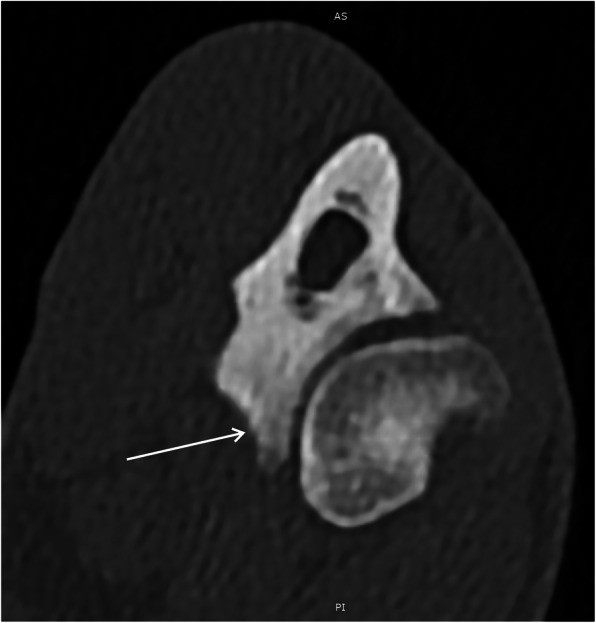
Frequency of CED-lesions (%) across tested population of dogs included in the study. INC R-U – radio-ulnar incongruence; INC H-U – humero-ulnar incongruence; FMCP – fragmented medial coronoid process; OCD – osteochondrosis dissecans within the medial compartment of humeral condyle coronoid process; MCD – medial coronoid disease without fragmented medial coronoid process (pathological cartilage and or subchondral bone); UAP – ununited anconeal process

The percentage distribution of CED lesions across the population of dogs included in the study is shown in Fig. 12.

One hundred and seventeen dogs out of 169 (69.23%) of dogs diagnosed with CED in our study were 2 years old or less. Several breeds, including Boxers, Dog de Bordeaux, American Staffordshire terriers and Mixed-breed dogs, were presented later in the course of their lifespan. Median age in these groups was: 6 years for boxers **(**mean 5.0 ± 3.0 years); 3.0 years for mixed-breed dogs (mean 4.5 ± 2.59 years); 4.75 years for American Staffordshire terriers (mean 5.16 ± 3.70 years) and 3.5 years for Dog de Bordeaux group (mean 3.16 ± 2.45 years).

The prevalence of CED components within breeds (along with the absolute number of dogs affected) and the occurrence of these pathologies in single-digit breeds are shown in Tables 6 and 7, respectively.

**Table 6:** Absolute number of dogs affected with various CED components within breeds

Breed	Left elbow joint						Right elbow joint					
	INC R-U	INC H-U	OCD	MCD	FMCP	UAP	INC R-U	INC H-U	OCD	MCD	FMCP	UAP
Labrador Retriever	9/43	21/43	4/43	19/43	17/43	0/43	10/43	21/43	5/43	13/43	21/43	0/43
German Shepherd Dog	18/40	31/40	0/40	13/40	12/40	6/40	19/40	32/40	1/40	14/35	17/40	6/40
Bernese Mountain dog	8/15	13/15	1/15	5/15	8/15	2/15	9/15	13/15	1/15	5/15	10/15	1/15
Chow Chow	3/6	6/6	2/6	0/6	6/6	0/6	2/6	6/6	2/6	0/6	6/6	0/6
Golden Retriever	0/5	2/5	2/5	1/5	0/5	0/5	1/5	3/5	2/5	2/5	0/5	0/5
Boxer	2/5	1/5	0/5	2/5	2/5	0/5	2/5	2/5	0/5	1/5	3/5	0/5
Cane Corso	2/5	4/5	0/5	0/5	3/5	1/5	1/5	5/5	0/5	0/5	3/5	2/5
Dog de Bordeaux	0/5	3/5	2/5	2/5	2/5	0/5	0/5	4/5	2/5	3/5	1/5	0/5
Rottweiler	1/4	3/4	1/4	2/4	1/4	0/4	1/4	4/4	1/4	4/4	0/4	0/4
White Swiss Shepherd	1/4	2/4	0/4	1/4	0/4	0/4	0/4	3/4	0/4	1/4	2/4	0/4
American Staff. Terrier	2/3	1/3	3/3	3/3	0/3	0/3	1/3	0/3	0/3	1/3	0/3	0/3
Bouvier des Flandres	2/3	3/3	1/3	1/3	2/3	0/3	1/3	2/3	2/3	2/3	1/3	0/3
Polish Tatra Sheepdog	1/3	2/3	1/3	0/3	2/3	0/3	1/3	2/3	0/3	1/3	1/3	0/3
English Bulldog	1/3	0/3	0/3	1/3	0/3	0/3	0/3	0/3	0/3	1/3	2/3	0/3
Eurasian Shepherd	0/2	1/2	0/2	1/2	0/2	0/2	1/2	2/2	0/2	1/2	0/2	1/2
Mix-breed	0/2	1/2	0/2	1/2	1/2	0/2	1/2	2/2	0/2	1/2	1/2	1/2
Caucasian Shepherd	0/2	2/2	0/2	0/2	1/2	1/2	0/2	2/2	0/2	2/2	0/2	1/2
Newfoundland	2/2	2/2	1/2	1/2	1/2	0/2	2/2	2/2	1/2	1/2	1/2	0/2
Argentinian Mastiff	1/2	2/2	0/2	2/2	0/2	0/2	1/2	2/2	0/2	2/2	0/2	0/2
Pitbull	0/2	0/2	0/2	0/2	0/2	0/2	0/2	0/2	0/2	1/2	0/2	0/2

*CED* canine elbow dysplasia; *INC R-U* radio-ulnar incongruence; *INC H-U* humero-ulnar incongruence; *FMCP* medial coronoid disease with fragmented medial coronoid process; *OCD* osteochondrosis dissecans within the medial compartment of humeral condyle coronoid process; *MCD* medial coronoid disease without fragmented medial coronoid process (pathological cartilage and or subchondral bone); *UAP* ununited anconeal process; 0 – no lesion

**Table 7:** Types of CED lesions in single-digit breed representants

	Left elbow joint						Right elbow joint					
	INC R-U	INC H-U	OCD	MCD	FMCP	UAP	INC R-U	INC H-U	OCD	MCD	FMCP	UAP
Flat Coated Retriever	0	1	0	1	0	0	0	0	0	0	0	0
Bullmastiff	1	1	0	0	0	0	1	1	0	1	0	0
Spanish Mastiff	0	1	0	0	1	0	0	1	0	0	1	0
Border Collie	0	0	0	0	0	0	0	1	0	1	0	0
Shar-pei	0	1	0	0	0	1	1	0	0	1	0	0
Giant Schnauzer	0	0	0	1	0	0	0	1	0	1	0	0
Spanish Alano	1	1	1	1	0	0	1	1	1	1	0	0
American Bully	0	1	0	1	0	0	1	0	0	1	0	0
Australian Cattle dog	1	0	0	1	0	0	0	0	0	0	0	0
Beagle	1	0	0	0	1	0	0	0	0	1	0	0
St. Bernard	0	1	0	0	0	1	0	1	0	0	0	1
Great Dane	0	1	0	0	1	0	0	0	0	1	0	0
Greater Swiss Mountain	0	0	0	1	0	0	0	1	0	1	0	0

*CED* canine elbow dysplasia; *INC R-U* radio-ulnar incongruence; *INC H-U* humero-ulnar incongruence; *FMCP* medial coronoid disease with fragmented medial coronoid process; *OCD* osteochondrosis dissecans within the medial compartment of humeral condyle coronoid process; *MCD* medial coronoid disease without fragmented medial coronoid process (pathological cartilage and or subchondral bone); *UAP* ununited anconeal process; “1 “lesion confirmed; “0 “– no lesion

## Discussion

Computed tomography imaging performed in a large cohort of CED – affected dogs allowed for assessment of prevalence of different CED components for each breed and across the whole population of dogs included in the present study. In accordance with previous findings, in the present study the elbow dysplasia was more common in males than in females [21].. It is hypothesized that sex distribution is associated with dominant inheritance within male linage [30]. The demographic data demonstrate inclusion of dogs of typical breeds (mostly large breeds) affected by CED, with wide range in age and severity of pathology. However, dogs presented with INC R-U and INC H-U were significantly younger than dogs without these CED components. In our study, similarly to data presented previously [31], lameness associated with CED was most common in Labrador Retriever and GSDs. In addition to CED, approximately 57% of dogs included in our study were diagnosed with concurrent OA of various degrees. Previous findings indicated that OA can develop as a primary lesion, although the presence of degenerative changes is often directly related to the development and progression of CED [32].

In the one-year prevalence study conducted by O’Neill et al. [26] the most common breeds among the incident elbow joint disease were Labrador Retriever (*n* = 189, 30.68%), GSD (*n* = 43, 6.98%), Staffordshire Bull Terrier (*n* = 32, 5.19%) and Rottweiler (*n* = 23, 3.73%), along with crossbred dogs (*n* = 100, 16.23%).

Our study showed that 11.6% of dogs were diagnosed with unilateral CED and 88.4% suffered from bilateral CED. The INC H-U was the most common finding, and approximately 40% of dogs diagnosed with INC H-U were also affected by MCD. Similar results were observed previously in Labrador Retrievers and Golden Retrievers [15].

Radio-ulnar incongruence was detected in 39.6% of dogs included in the present study, and approximately 50% of these dogs were also affected by FMCP. Previous study conducted by Eljack et al. (2013) showed, that up to 60% of dogs diagnosed with INC R-U were simultaneously affected by MCD, which complies with our findings [33]. Interestingly, 100% of the Chow Chow’s included in the present study (*n* = 6) were simultaneously affected by INC H-U and FMCP. This may be associated with generally higher heritability values for this breed as has been shown previously [1]. Osteochondritis dissecans was detected in 11.24% of dogs and was always accompanied by other CED lesions. In our study, the least common lesion found was UAP, which was present in 9.46% of dogs. Similar low prevalence of OCD and UAP was reported previously [9]. Simultaneous occurrence of FCMP and UAP was previously reported to be not common findings [34–36]. In the present study similar results were obtained. The simultaneous presence of FMCP and UAP was observed only in 3 dogs (GSD, *Bernese Mountain Dog*, Cane corso). Interestingly, in the present study several breeds with CED – associated lameness were older at first diagnosis (Boxers, Dog de Bordeaux, American Staffordshire Terriers and crossbreed dogs). However, we cannot present data on the time of first appearance of clinical signs in the tested dogs, which is one of the limitations of our study and should be taken into account when interpreting the results. Furthermore, due to the small number of dogs from breeds that represented late-onset CED in our study (5 Boxers, 5 Dog de Bordeaux, 3 American Staffordshire Terriers and 2 crossbreed dogs) more research is needed to confirm these findings. The relationship between age and presence of some components of CED has been reported previously [37]; however, data describing specific breeds are limited. In a study by Vermote et al. [37], patients older than 6 years presenting for initial arthroscopic treatment of MCD represented only 12% of dogs (77/660). From dogs older than 6 years included in the mentioned study, 13 out of 16 were Labrador Retrievers, 3 out of 4 were GSDs, 5 out of 6 were Golden Retrievers and 6 out of 8 were mixed-breed dogs. According to O’Neill et al. [26] the median age (in years) at first diagnosis for breeds with over 20 incident was: Labrador Retriever 6.42 (2.50–9.08, *n* = 188), German Shepherd Dog 5.64 (0.80–7.77, *n =* 42), Staffordshire Bull Terrier 8.02 (3.16–10.87, *n =* 32), Rottweiler 7.20 (1.47–8.17, *n =* 23), English Springer Spaniel 7.00 (1.77–12.27, *n =* 21), Golden Retriever 9.75 (5.27–11.65, *n =* 21) and crossbred dogs 7.65 (3.39–10.95, *n =* 100). However, all dogs with a diagnosis of elbow joint disease made by veterinarians were included in above mentioned study.

## Conclusions

INC H-U, FMCP and MCD were among the most frequently found components of CED (in 129, 92 and 89 dogs, respectively) in the present study. OCD and UAP were the least frequently diagnosed pathologies (in 19 and 16 dogs, respectively). 69.23% of dogs diagnosed with CED were young (≤ 2 years). Dogs diagnosed with INC R-U were significantly younger than dogs without this component of CED. A similar result was observed for INC H-U. Boxers, Dog de Bordeaux, American Staffordshire terriers and mixed-breed dogs were diagnosed later in life than the other breeds. OA of varying severity was found in 72.18% of dogs. Males accounted for more than 75% of the study population.

## Data Availability

The data used to support the findings of this study are available from the corresponding author upon reasonable request.

## Abbreviations

3D MPR: 3D Multiplanar reconstruction
CED: Canine elbow dysplasia
CT: Computed tomography
FMCP: Fragmented medial coronoid process (as a medial coronoid disease with fragmented medial coronoid process)
IEWG: International elbow work group guidelines
INC H-U: Humero-ulnar incongruence
INC R-U: Radio-ulnar incongruence
MCD: Medial coronoid disease (without fragmented medial coronoid process)
PANOS: Panosteitis
OA: Osteoarthritis
OC: Osteochondrosis
OCD: Osteochondrosis dissecans
UAP: Ununited anconeal process
